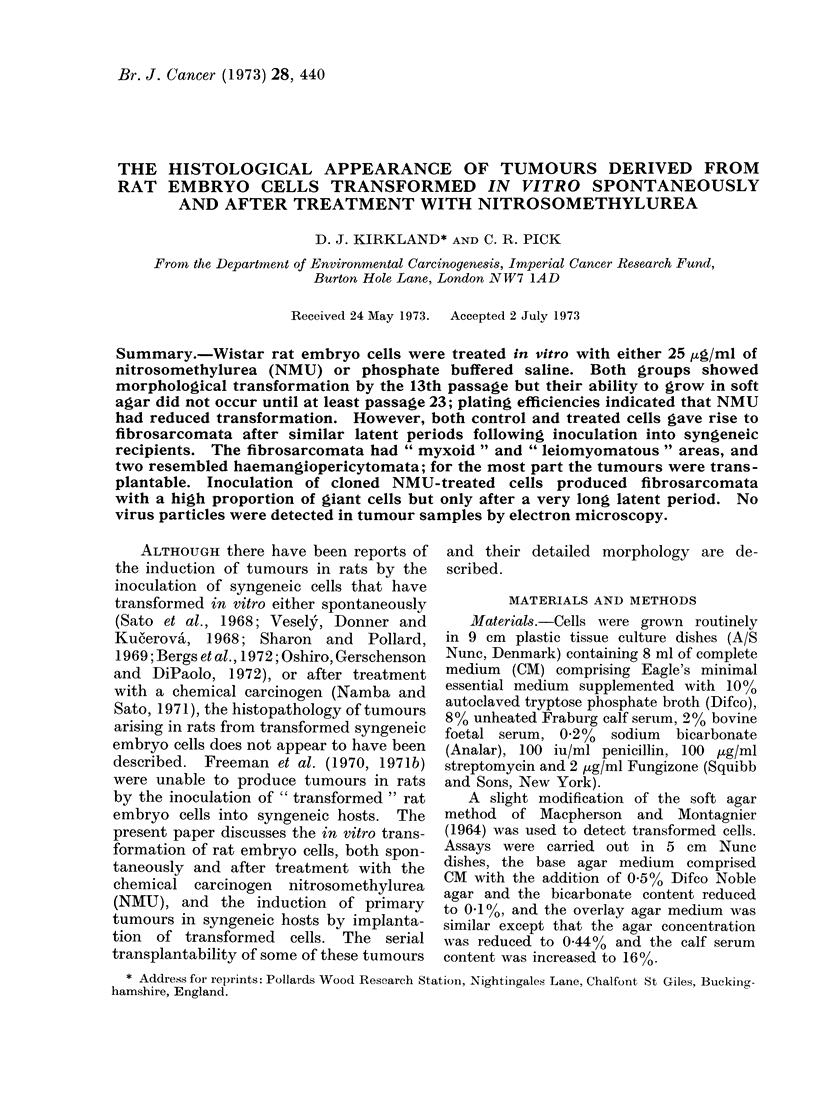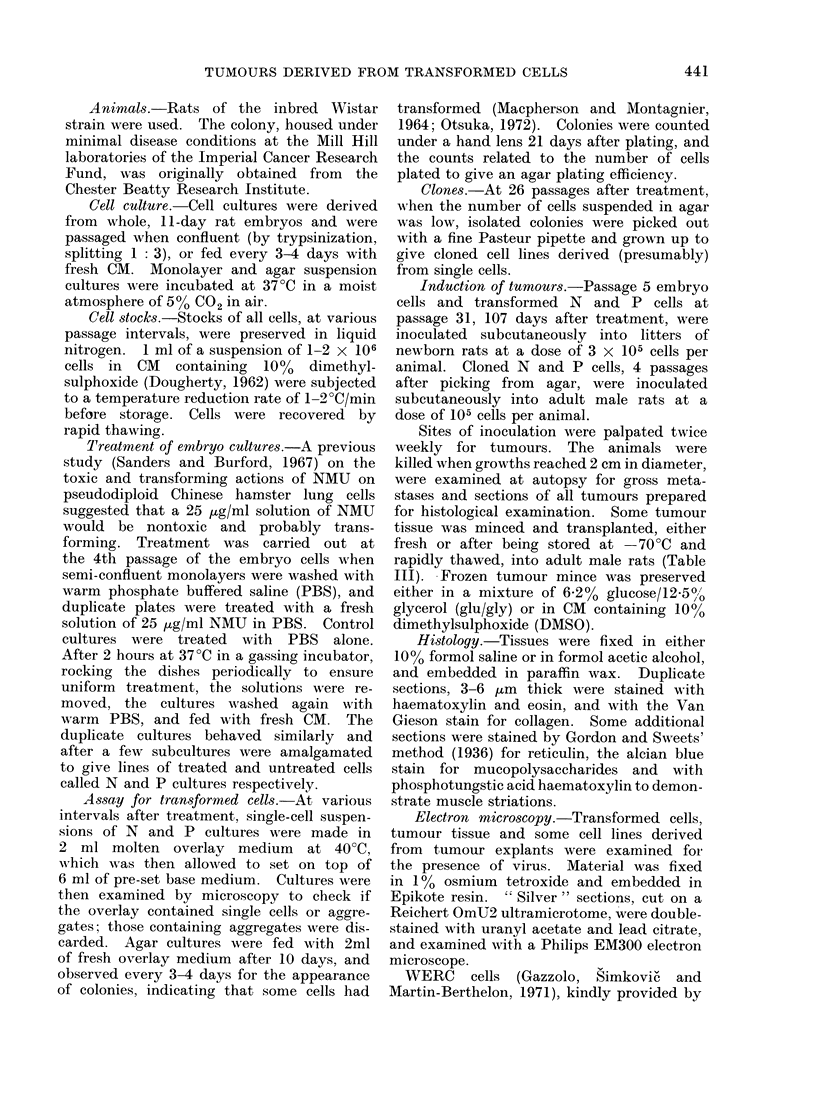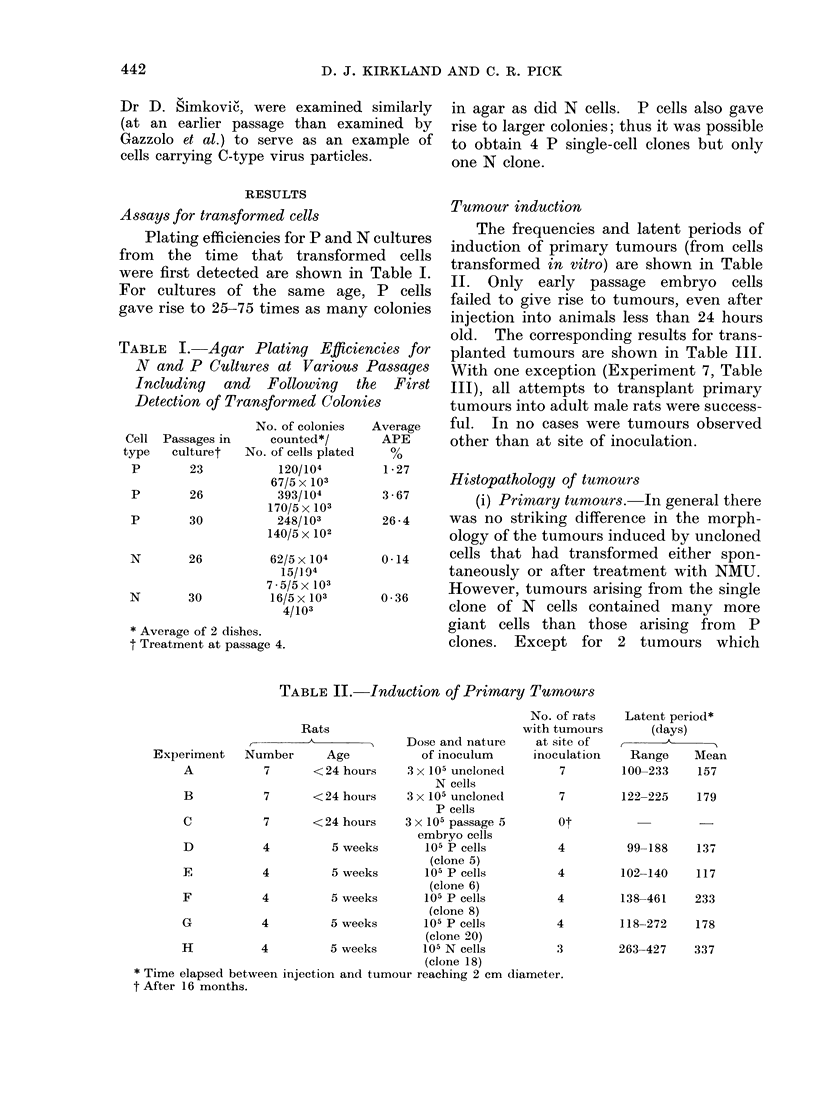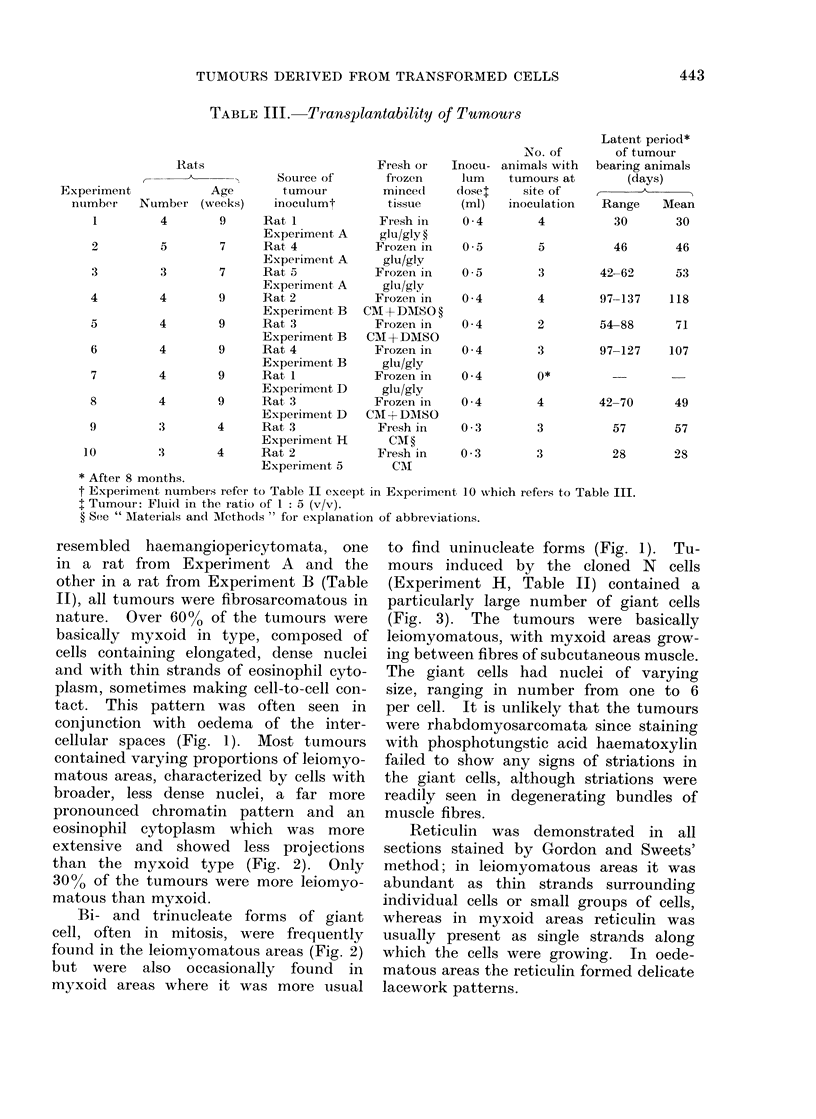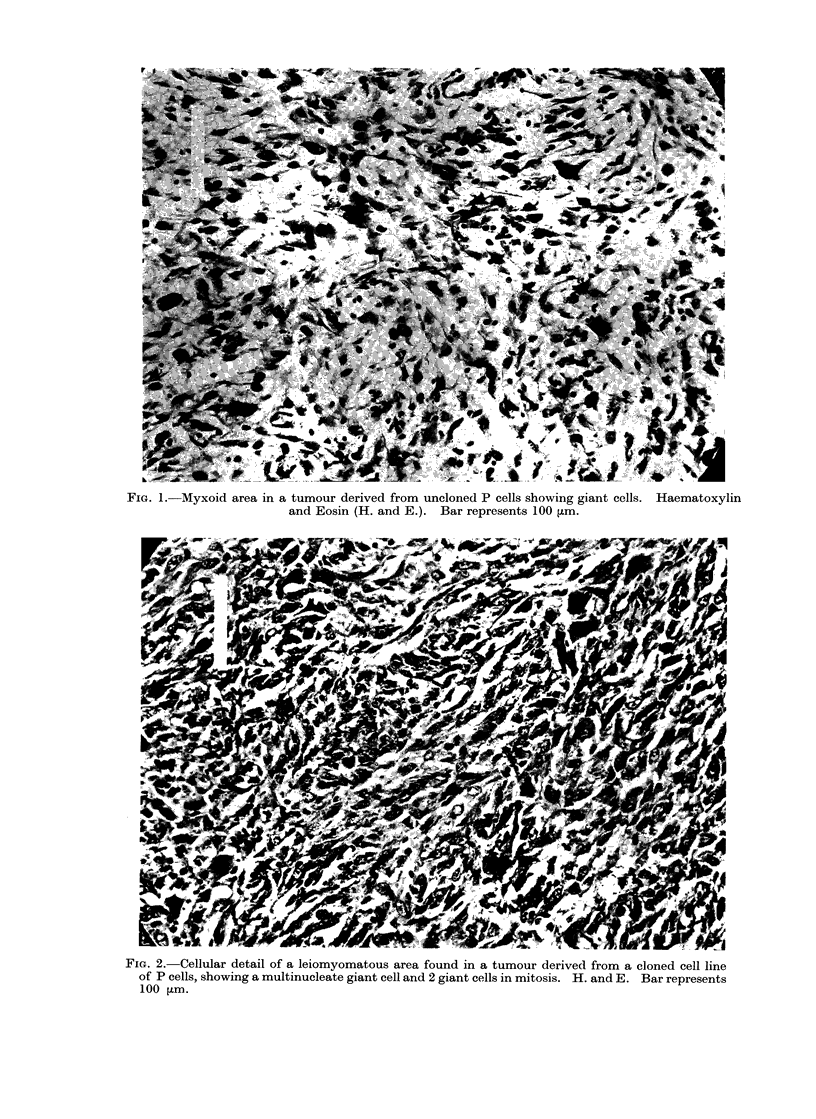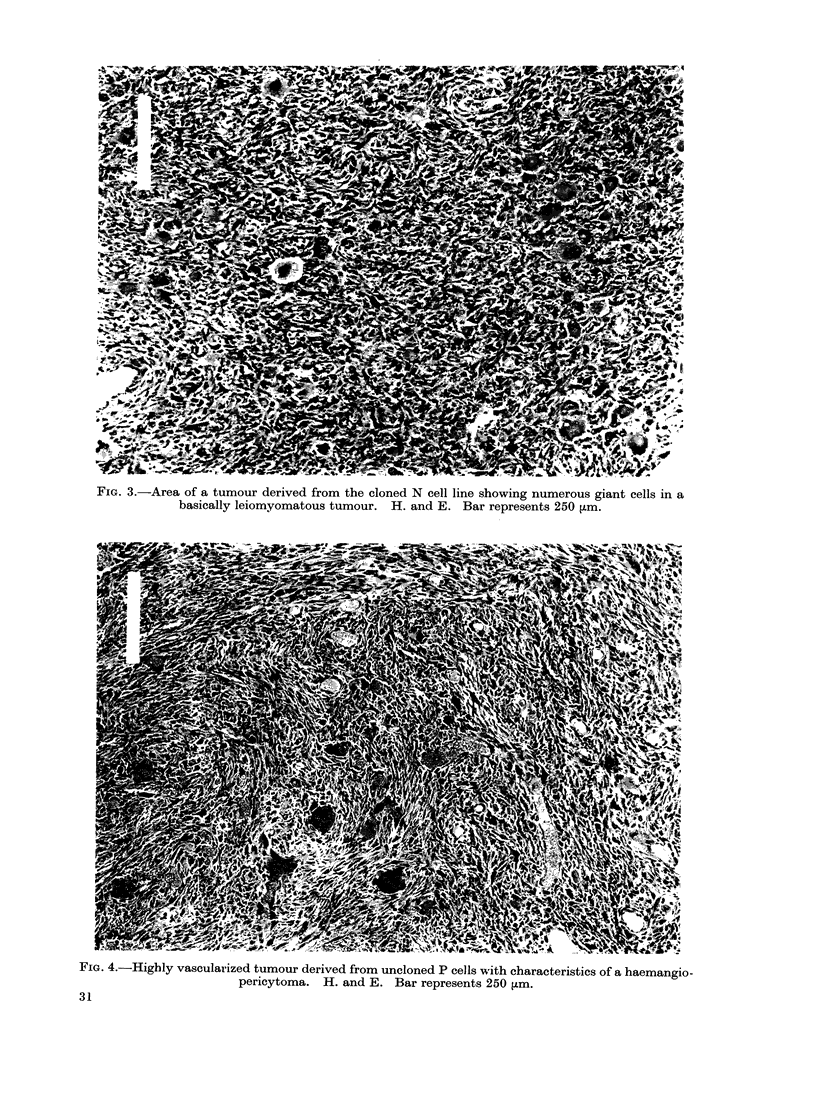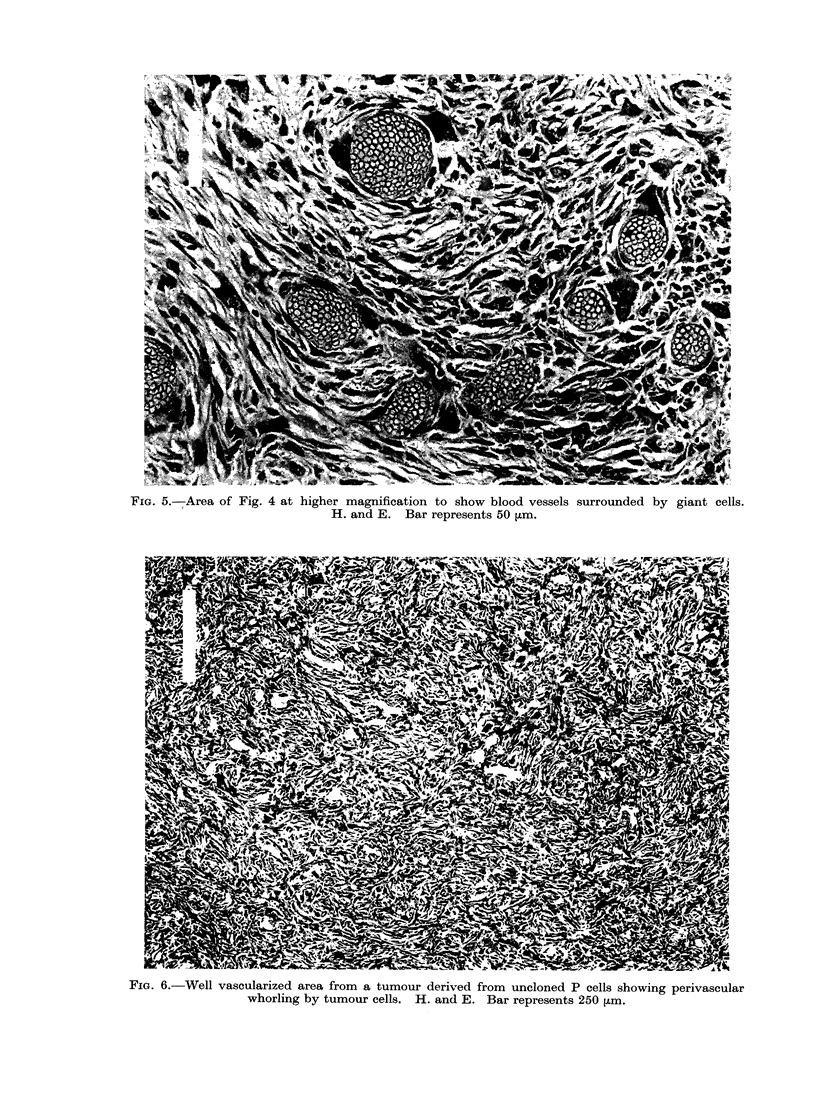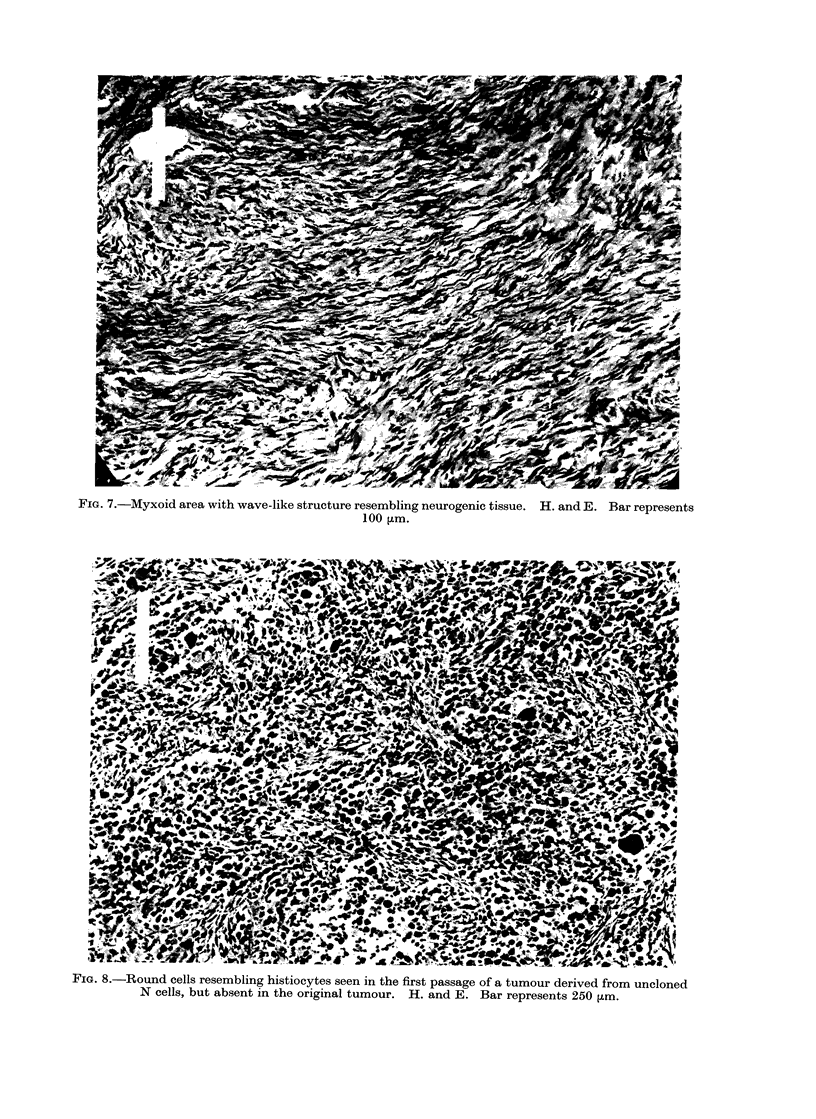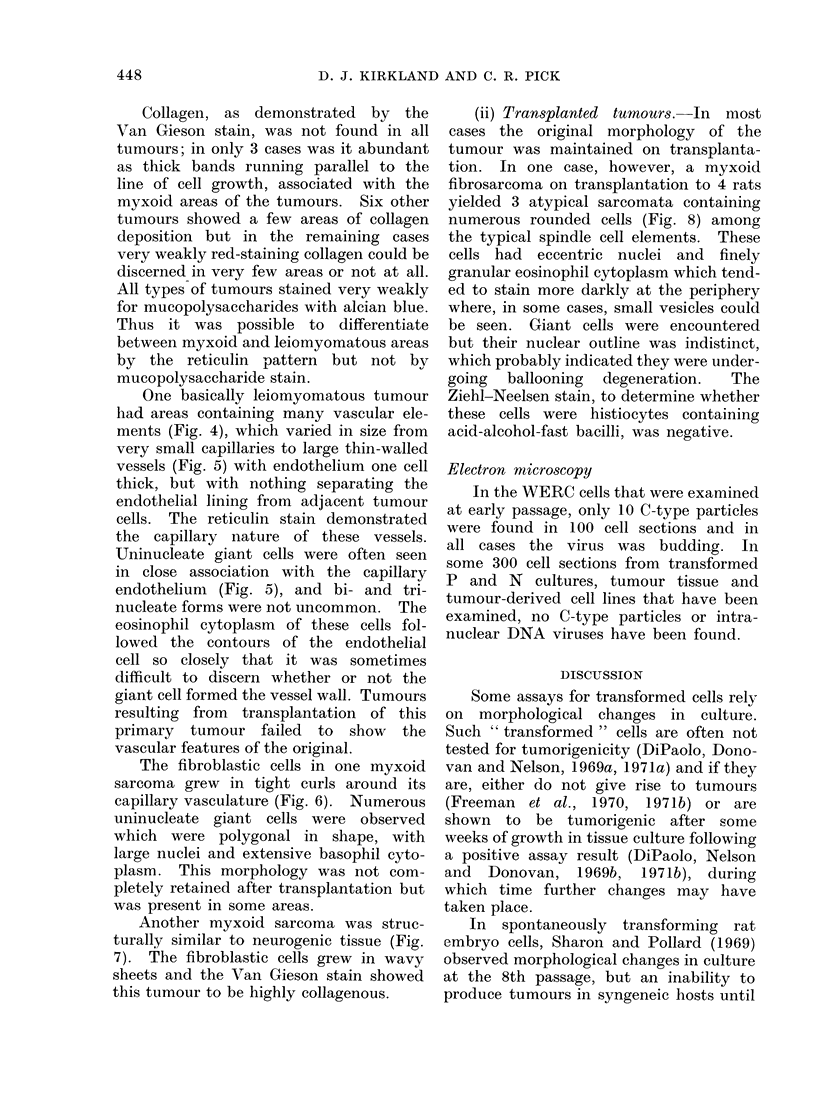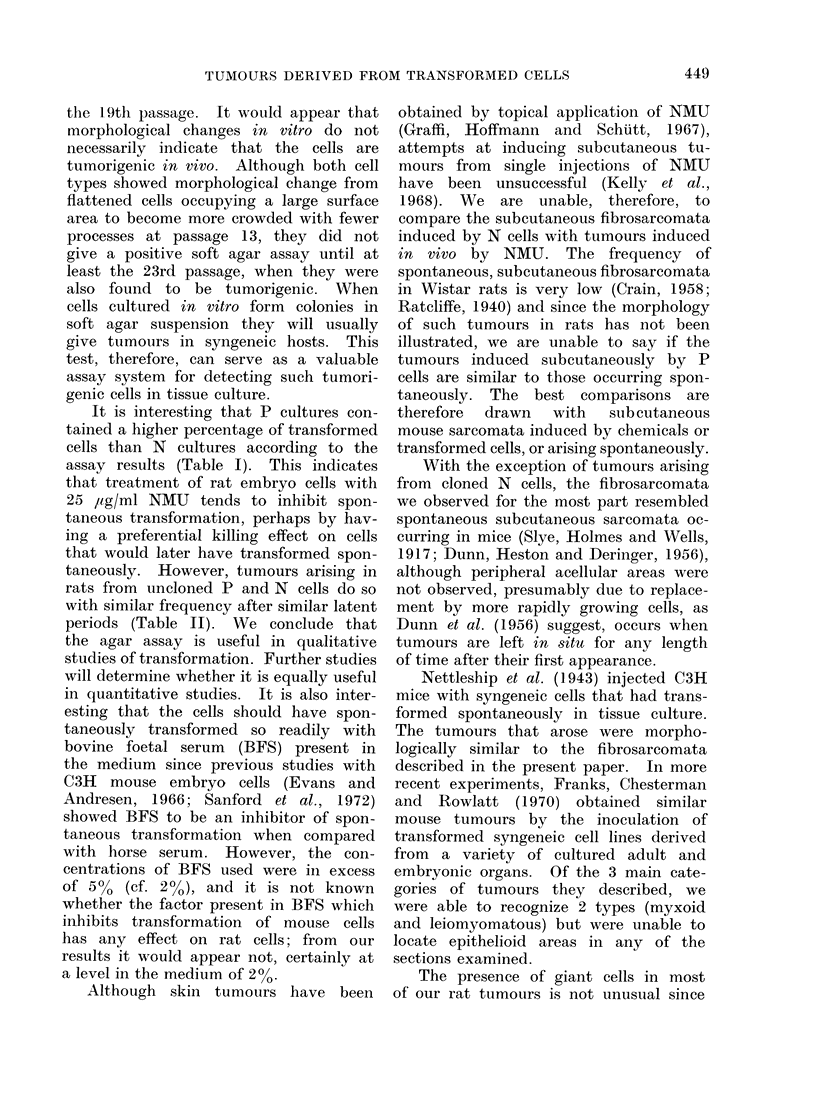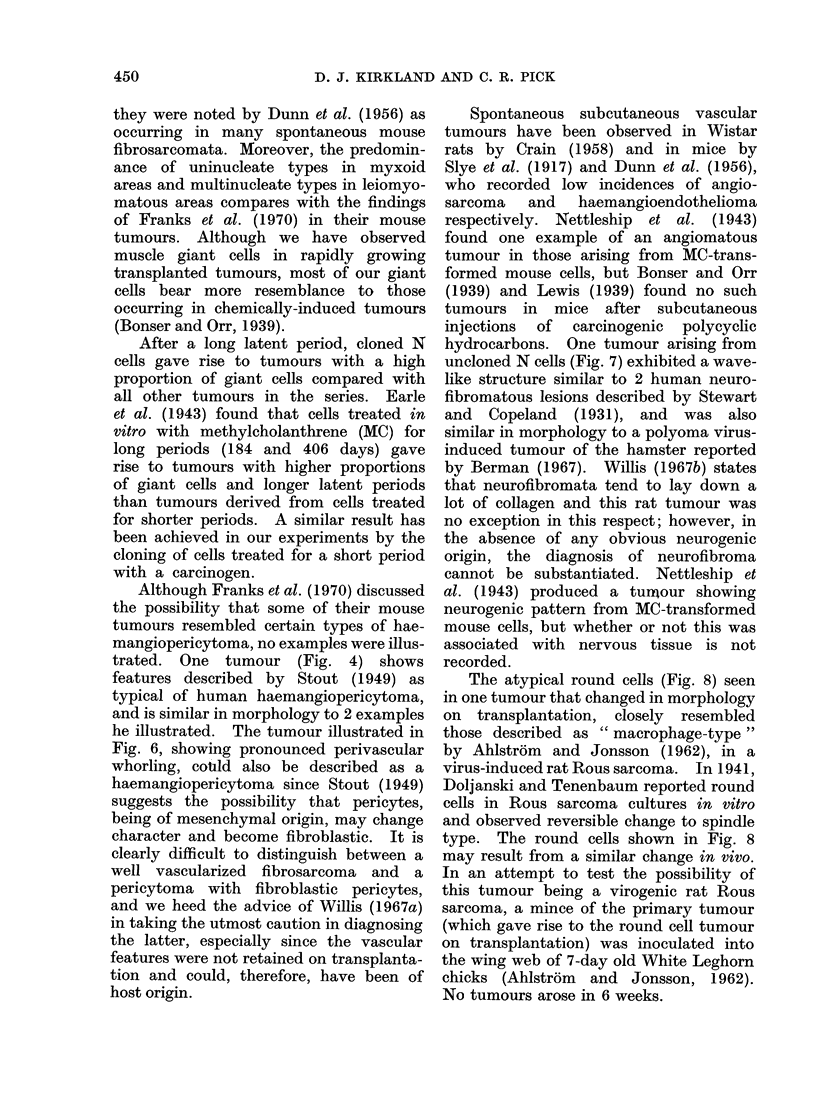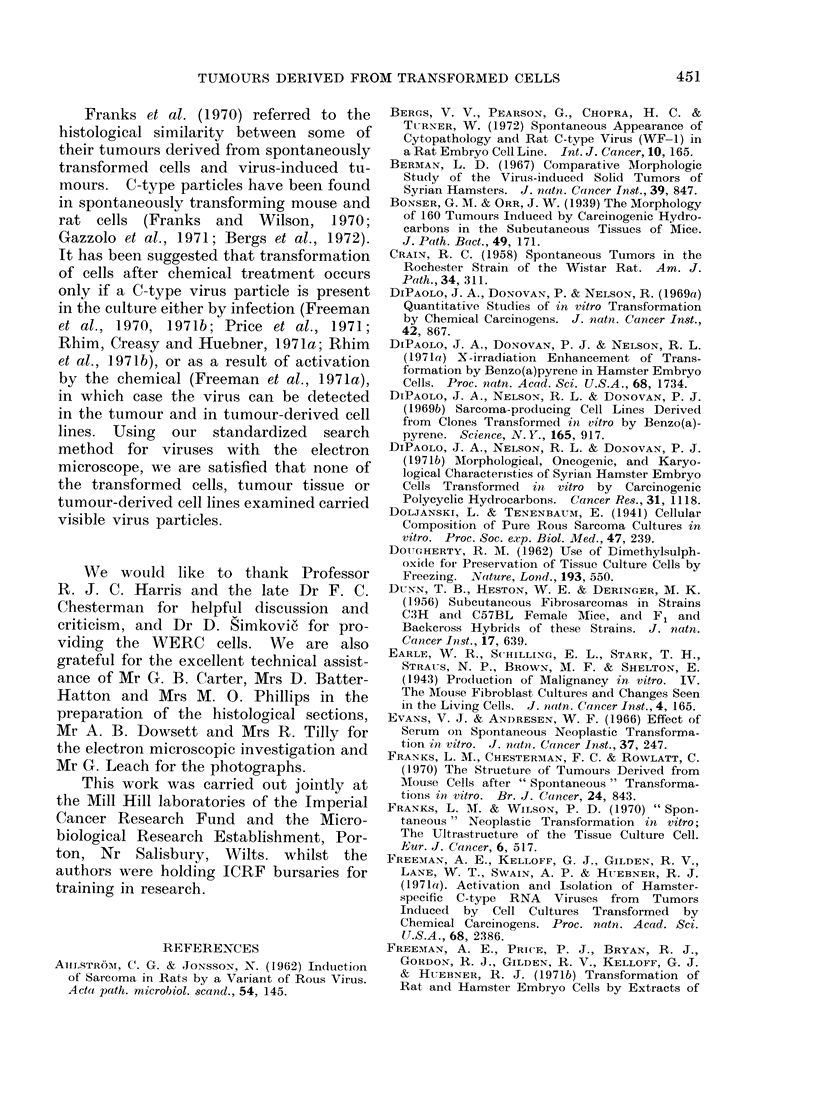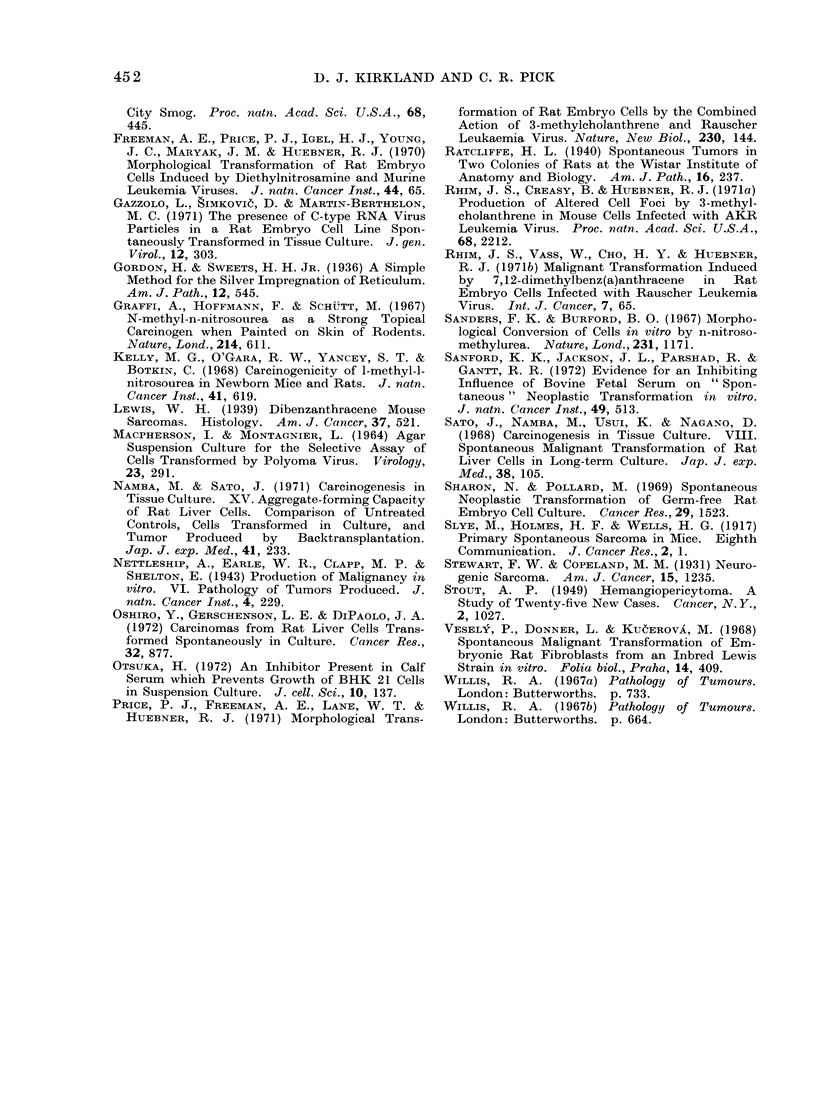# The Histological Appearance of Tumours Derived from Rat Embryo Cells Transformed in vitro Spontaneously and After Treatment with Nitrosomethylurea

**DOI:** 10.1038/bjc.1973.170

**Published:** 1973-11

**Authors:** D. J. Kirkland, C. R. Pick

## Abstract

**Images:**


*Br. J. Cancer* (1973) **28**, 440

### THE HISTOLOGICAL APPEARANCE OF TUMOURS DERIVED FROM RAT EMBRYO CELLS TRANSFORMED *IN VITRO* SPONTANEOUSLY AND AFTER TREATMENT WITH NITROSOMETHYLUREA

D. J. KIRKLAND* AND C. R. PICK

*From the Department of Environmental Carcinogenesis, Imperial Cancer Research Fund, Burton Hole Lane, London NW7 1AD*

Received 24 May 1973. Accepted 2 July 1973

**Summary.—Wistar rat embryo cells were treated *in vitro* with either 25 μg/ml of nitrosomethylurea (NMU) or phosphate buffered saline. Both groups showed morphological transformation by the 13th passage but their ability to grow in soft agar did not occur until at least passage 23; plating efficiencies indicated that NMU had reduced transformation. However, both control and treated cells gave rise to fibrosarcomata after similar latent periods following inoculation into syngeneic recipients. The fibrosarcomata had " myxoid " and " leiomyomatous " areas, and two resembled haemangiopericytomata; for the most part the tumours were transplantable. Inoculation of cloned NMU-treated cells produced fibrosarcomata with a high proportion of giant cells but only after a very long latent period. No virus particles were detected in tumour samples by electron microscopy.**

ALTHOUGH there have been reports of the induction of tumours in rats by the inoculation of syngeneic cells that have transformed *in vitro* either spontaneously (Sato *et al.*, 1968; Veselý, Donner and Kučerová, 1968; Sharon and Pollard, 1969; Bergs *et al.*, 1972; Oshiro, Gerschenson and DiPaolo, 1972), or after treatment with a chemical carcinogen (Namba and Sato, 1971), the histopathology of tumours arising in rats from transformed syngeneic embryo cells does not appear to have been described. Freeman *et al.* (1970, 1971*b*) were unable to produce tumours in rats by the inoculation of " transformed " rat embryo cells into syngeneic hosts. The present paper discusses the *in vitro* transformation of rat embryo cells, both spontaneously and after treatment with the chemical carcinogen nitrosomethylurea (NMU), and the induction of primary tumours in syngeneic hosts by implantation of transformed cells. The serial transplantability of some of these tumours and their detailed morphology are described.

### MATERIALS AND METHODS

*Materials.*—Cells were grown routinely in 9 cm plastic tissue culture dishes (A/S Nunc, Denmark) containing 8 ml of complete medium (CM) comprising Eagle's minimal essential medium supplemented with 10% autoclaved tryptose phosphate broth (Difco), 8% unheated Fraburg calf serum, 2% bovine foetal serum, 0·2% sodium bicarbonate (Analar), 100 iu/ml penicillin, 100 μg/ml streptomycin and 2 μg/ml Fungizone (Squibb and Sons, New York).

A slight modification of the soft agar method of Macpherson and Montagnier (1964) was used to detect transformed cells. Assays were carried out in 5 cm Nunc dishes, the base agar medium comprised CM with the addition of 0·5% Difco Noble agar and the bicarbonate content reduced to 0·1%, and the overlay agar medium was similar except that the agar concentration was reduced to 0·44% and the calf serum content was increased to 16%.

* Address for reprints: Pollards Wood Research Station, Nightingales Lane, Chalfont St Giles, Buckinghamshire, England.

*Animals.*—Rats of the inbred Wistar strain were used. The colony, housed under minimal disease conditions at the Mill Hill laboratories of the Imperial Cancer Research Fund, was originally obtained from the Chester Beatty Research Institute.

*Cell culture.*—Cell cultures were derived from whole, 11-day rat embryos and were passaged when confluent (by trypsinization, splitting 1 : 3), or fed every 3–4 days with fresh CM. Monolayer and agar suspension cultures were incubated at 37°C in a moist atmosphere of 5% $CO_2$ in air.

*Cell stocks.*—Stocks of all cells, at various passage intervals, were preserved in liquid nitrogen. 1 ml of a suspension of 1–2 $\times$ $10^6$ cells in CM containing 10% dimethylsulphoxide (Dougherty, 1962) were subjected to a temperature reduction rate of 1–2°C/min before storage. Cells were recovered by rapid thawing.

*Treatment of embryo cultures.*—A previous study (Sanders and Burford, 1967) on the toxic and transforming actions of NMU on pseudodiploid Chinese hamster lung cells suggested that a 25 $\mu$g/ml solution of NMU would be nontoxic and probably transforming. Treatment was carried out at the 4th passage of the embryo cells when semi-confluent monolayers were washed with warm phosphate buffered saline (PBS), and duplicate plates were treated with a fresh solution of 25 $\mu$g/ml NMU in PBS. Control cultures were treated with PBS alone. After 2 hours at 37°C in a gassing incubator, rocking the dishes periodically to ensure uniform treatment, the solutions were removed, the cultures washed again with warm PBS, and fed with fresh CM. The duplicate cultures behaved similarly and after a few subcultures were amalgamated to give lines of treated and untreated cells called N and P cultures respectively.

*Assay for transformed cells.*—At various intervals after treatment, single-cell suspensions of N and P cultures were made in 2 ml molten overlay medium at 40°C, which was then allowed to set on top of 6 ml of pre-set base medium. Cultures were then examined by microscopy to check if the overlay contained single cells or aggregates; those containing aggregates were discarded. Agar cultures were fed with 2ml of fresh overlay medium after 10 days, and observed every 3–4 days for the appearance of colonies, indicating that some cells had transformed (Macpherson and Montagnier, 1964; Otsuka, 1972). Colonies were counted under a hand lens 21 days after plating, and the counts related to the number of cells plated to give an agar plating efficiency.

*Clones.*—At 26 passages after treatment, when the number of cells suspended in agar was low, isolated colonies were picked out with a fine Pasteur pipette and grown up to give cloned cell lines derived (presumably) from single cells.

*Induction of tumours.*—Passage 5 embryo cells and transformed N and P cells at passage 31, 107 days after treatment, were inoculated subcutaneously into litters of newborn rats at a dose of 3 $\times$ $10^5$ cells per animal. Cloned N and P cells, 4 passages after picking from agar, were inoculated subcutaneously into adult male rats at a dose of $10^5$ cells per animal.

Sites of inoculation were palpated twice weekly for tumours. The animals were killed when growths reached 2 cm in diameter, were examined at autopsy for gross metastases and sections of all tumours prepared for histological examination. Some tumour tissue was minced and transplanted, either fresh or after being stored at −70°C and rapidly thawed, into adult male rats (Table III). Frozen tumour mince was preserved either in a mixture of 6·2% glucose/12·5% glycerol (glu/gly) or in CM containing 10% dimethylsulphoxide (DMSO).

*Histology.*—Tissues were fixed in either 10% formol saline or in formol acetic alcohol, and embedded in paraffin wax. Duplicate sections, 3–6 $\mu$m thick were stained with haematoxylin and eosin, and with the Van Gieson stain for collagen. Some additional sections were stained by Gordon and Sweets' method (1936) for reticulin, the alcian blue stain for mucopolysaccharides and with phosphotungstic acid haematoxylin to demonstrate muscle striations.

*Electron microscopy.*—Transformed cells, tumour tissue and some cell lines derived from tumour explants were examined for the presence of virus. Material was fixed in 1% osmium tetroxide and embedded in Epikote resin. "Silver" sections, cut on a Reichert OmU2 ultramicrotome, were double-stained with uranyl acetate and lead citrate, and examined with a Philips EM300 electron microscope.

WERC cells (Gazzolo, Šimkovič and Martin-Berthelon, 1971), kindly provided by

Dr D. Šimkovič, were examined similarly (at an earlier passage than examined by Gazzolo *et al.*) to serve as an example of cells carrying C-type virus particles.

RESULTS

*Assays for transformed cells*

Plating efficiencies for P and N cultures from the time that transformed cells were first detected are shown in Table I. For cultures of the same age, P cells gave rise to 25–75 times as many colonies in agar as did N cells. P cells also gave rise to larger colonies; thus it was possible to obtain 4 P single-cell clones but only one N clone.

TABLE I.—*Agar Plating Efficiencies for N and P Cultures at Various Passages Including and Following the First Detection of Transformed Colonies*

| Cell type | Passages in culture† | No. of colonies counted*/ No. of cells plated | Average APE % |
|---|---|---|---|
| P | 23 | $120/10^4$ | 1·27 |
| | | $67/5\times10^3$ | |
| P | 26 | $393/10^4$ | 3·67 |
| | | $170/5\times10^3$ | |
| P | 30 | $248/10^3$ | 26·4 |
| | | $140/5\times10^2$ | |
| N | 26 | $62/5\times10^4$ | 0·14 |
| | | $15/10^4$ | |
| | | $7\cdot5/5\times10^3$ | |
| N | 30 | $16/5\times10^3$ | 0·36 |
| | | $4/10^3$ | |

* Average of 2 dishes.

† Treatment at passage 4.

*Tumour induction*

The frequencies and latent periods of induction of primary tumours (from cells transformed *in vitro*) are shown in Table II. Only early passage embryo cells failed to give rise to tumours, even after injection into animals less than 24 hours old. The corresponding results for transplanted tumours are shown in Table III. With one exception (Experiment 7, Table III), all attempts to transplant primary tumours into adult male rats were successful. In no cases were tumours observed other than at site of inoculation.

*Histopathology of tumours*

(i) *Primary tumours.*—In general there was no striking difference in the morphology of the tumours induced by uncloned cells that had transformed either spontaneously or after treatment with NMU. However, tumours arising from the single clone of N cells contained many more giant cells than those arising from P clones. Except for 2 tumours which

TABLE II.—*Induction of Primary Tumours*

| Experiment | Rats | | Dose and nature of inoculum | No. of rats with tumours at site of inoculation | Latent period* (days) | |
|---|---|---|---|---|---|---|
| | Number | Age | | | Range | Mean |
| A | 7 | <24 hours | $3\times10^5$ uncloned N cells | 7 | 100–233 | 157 |
| B | 7 | <24 hours | $3\times10^5$ uncloned P cells | 7 | 122–225 | 179 |
| C | 7 | <24 hours | $3\times10^5$ passage 5 embryo cells | 0† | — | — |
| D | 4 | 5 weeks | $10^5$ P cells (clone 5) | 4 | 99–188 | 137 |
| E | 4 | 5 weeks | $10^5$ P cells (clone 6) | 4 | 102–140 | 117 |
| F | 4 | 5 weeks | $10^5$ P cells (clone 8) | 4 | 138–461 | 233 |
| G | 4 | 5 weeks | $10^5$ P cells (clone 20) | 4 | 118–272 | 178 |
| H | 4 | 5 weeks | $10^5$ N cells (clone 18) | 3 | 263–427 | 337 |

* Time elapsed between injection and tumour reaching 2 cm diameter.
† After 16 months.

TABLE III.—*Transplantability of Tumours*

| Experiment number | Rats: Number | Rats: Age (weeks) | Source of tumour inoculum† | Fresh or frozen minced tissue | Inoculum dose‡ (ml) | No. of animals with tumours at site of inoculation | Latent period* of tumour bearing animals (days): Range | Mean |
|---|---|---|---|---|---|---|---|---|
| 1 | 4 | 9 | Rat 1 Experiment A | Fresh in glu/gly § | 0·4 | 4 | 30 | 30 |
| 2 | 5 | 7 | Rat 4 Experiment A | Frozen in glu/gly | 0·5 | 5 | 46 | 46 |
| 3 | 3 | 7 | Rat 5 Experiment A | Frozen in glu/gly | 0·5 | 3 | 42–62 | 53 |
| 4 | 4 | 9 | Rat 2 Experiment B | Frozen in CM+DMSO § | 0·4 | 4 | 97–137 | 118 |
| 5 | 4 | 9 | Rat 3 Experiment B | Frozen in CM+DMSO | 0·4 | 2 | 54–88 | 71 |
| 6 | 4 | 9 | Rat 4 Experiment B | Frozen in glu/gly | 0·4 | 3 | 97–127 | 107 |
| 7 | 4 | 9 | Rat 1 Experiment D | Frozen in glu/gly | 0·4 | 0* | — | — |
| 8 | 4 | 9 | Rat 3 Experiment D | Frozen in CM+DMSO | 0·4 | 4 | 42–70 | 49 |
| 9 | 3 | 4 | Rat 3 Experiment H | Fresh in CM § | 0·3 | 3 | 57 | 57 |
| 10 | 3 | 4 | Rat 2 Experiment 5 | Fresh in CM | 0·3 | 3 | 28 | 28 |

* After 8 months.
† Experiment numbers refer to Table II except in Experiment 10 which refers to Table III.
‡ Tumour: Fluid in the ratio of 1 : 5 (v/v).
§ See " Materials and Methods " for explanation of abbreviations.

resembled haemangiopericytomata, one in a rat from Experiment A and the other in a rat from Experiment B (Table II), all tumours were fibrosarcomatous in nature. Over 60% of the tumours were basically myxoid in type, composed of cells containing elongated, dense nuclei and with thin strands of eosinophil cytoplasm, sometimes making cell-to-cell contact. This pattern was often seen in conjunction with oedema of the intercellular spaces (Fig. 1). Most tumours contained varying proportions of leiomyomatous areas, characterized by cells with broader, less dense nuclei, a far more pronounced chromatin pattern and an eosinophil cytoplasm which was more extensive and showed less projections than the myxoid type (Fig. 2). Only 30% of the tumours were more leiomyomatous than myxoid.

Bi- and trinucleate forms of giant cell, often in mitosis, were frequently found in the leiomyomatous areas (Fig. 2) but were also occasionally found in myxoid areas where it was more usual to find uninucleate forms (Fig. 1). Tumours induced by the cloned N cells (Experiment H, Table II) contained a particularly large number of giant cells (Fig. 3). The tumours were basically leiomyomatous, with myxoid areas growing between fibres of subcutaneous muscle. The giant cells had nuclei of varying size, ranging in number from one to 6 per cell. It is unlikely that the tumours were rhabdomyosarcomata since staining with phosphotungstic acid haematoxylin failed to show any signs of striations in the giant cells, although striations were readily seen in degenerating bundles of muscle fibres.

Reticulin was demonstrated in all sections stained by Gordon and Sweets' method; in leiomyomatous areas it was abundant as thin strands surrounding individual cells or small groups of cells, whereas in myxoid areas reticulin was usually present as single strands along which the cells were growing. In oedematous areas the reticulin formed delicate lacework patterns.

FIG. 1.—Myxoid area in a tumour derived from uncloned P cells showing giant cells. Haematoxylin and Eosin (H. and E.). Bar represents 100 μm.

FIG. 2.—Cellular detail of a leiomyomatous area found in a tumour derived from a cloned cell line of P cells, showing a multinucleate giant cell and 2 giant cells in mitosis. H. and E. Bar represents 100 μm.

FIG. 3.—Area of a tumour derived from the cloned N cell line showing numerous giant cells in a basically leiomyomatous tumour. H. and E. Bar represents 250 μm.

FIG. 4.—Highly vascularized tumour derived from uncloned P cells with characteristics of a haemangio-pericytoma. H. and E. Bar represents 250 μm.

FIG. 5.—Area of Fig. 4 at higher magnification to show blood vessels surrounded by giant cells. H. and E. Bar represents 50 μm.

FIG. 6.—Well vascularized area from a tumour derived from uncloned P cells showing perivascular whorling by tumour cells. H. and E. Bar represents 250 μm.

FIG. 7.—Myxoid area with wave-like structure resembling neurogenic tissue. H. and E. Bar represents 100 μm.

FIG. 8.—Round cells resembling histiocytes seen in the first passage of a tumour derived from uncloned N cells, but absent in the original tumour. H. and E. Bar represents 250 μm.

Collagen, as demonstrated by the Van Gieson stain, was not found in all tumours; in only 3 cases was it abundant as thick bands running parallel to the line of cell growth, associated with the myxoid areas of the tumours. Six other tumours showed a few areas of collagen deposition but in the remaining cases very weakly red-staining collagen could be discerned in very few areas or not at all. All types of tumours stained very weakly for mucopolysaccharides with alcian blue. Thus it was possible to differentiate between myxoid and leiomyomatous areas by the reticulin pattern but not by mucopolysaccharide stain.

One basically leiomyomatous tumour had areas containing many vascular elements (Fig. 4), which varied in size from very small capillaries to large thin-walled vessels (Fig. 5) with endothelium one cell thick, but with nothing separating the endothelial lining from adjacent tumour cells. The reticulin stain demonstrated the capillary nature of these vessels. Uninucleate giant cells were often seen in close association with the capillary endothelium (Fig. 5), and bi- and trinucleate forms were not uncommon. The eosinophil cytoplasm of these cells followed the contours of the endothelial cell so closely that it was sometimes difficult to discern whether or not the giant cell formed the vessel wall. Tumours resulting from transplantation of this primary tumour failed to show the vascular features of the original.

The fibroblastic cells in one myxoid sarcoma grew in tight curls around its capillary vasculature (Fig. 6). Numerous uninucleate giant cells were observed which were polygonal in shape, with large nuclei and extensive basophil cytoplasm. This morphology was not completely retained after transplantation but was present in some areas.

Another myxoid sarcoma was structurally similar to neurogenic tissue (Fig. 7). The fibroblastic cells grew in wavy sheets and the Van Gieson stain showed this tumour to be highly collagenous.

(ii) *Transplanted tumours.*—In most cases the original morphology of the tumour was maintained on transplantation. In one case, however, a myxoid fibrosarcoma on transplantation to 4 rats yielded 3 atypical sarcomata containing numerous rounded cells (Fig. 8) among the typical spindle cell elements. These cells had eccentric nuclei and finely granular eosinophil cytoplasm which tended to stain more darkly at the periphery where, in some cases, small vesicles could be seen. Giant cells were encountered but their nuclear outline was indistinct, which probably indicated they were undergoing ballooning degeneration. The Ziehl–Neelsen stain, to determine whether these cells were histiocytes containing acid-alcohol-fast bacilli, was negative.

*Electron microscopy*

In the WERC cells that were examined at early passage, only 10 C-type particles were found in 100 cell sections and in all cases the virus was budding. In some 300 cell sections from transformed P and N cultures, tumour tissue and tumour-derived cell lines that have been examined, no C-type particles or intranuclear DNA viruses have been found.

### DISCUSSION

Some assays for transformed cells rely on morphological changes in culture. Such " transformed " cells are often not tested for tumorigenicity (DiPaolo, Donovan and Nelson, 1969*a*, 1971*a*) and if they are, either do not give rise to tumours (Freeman *et al.*, 1970, 1971*b*) or are shown to be tumorigenic after some weeks of growth in tissue culture following a positive assay result (DiPaolo, Nelson and Donovan, 1969*b*, 1971*b*), during which time further changes may have taken place.

In spontaneously transforming rat embryo cells, Sharon and Pollard (1969) observed morphological changes in culture at the 8th passage, but an inability to produce tumours in syngeneic hosts until the 19th passage. It would appear that morphological changes *in vitro* do not necessarily indicate that the cells are tumorigenic *in vivo*. Although both cell types showed morphological change from flattened cells occupying a large surface area to become more crowded with fewer processes at passage 13, they did not give a positive soft agar assay until at least the 23rd passage, when they were also found to be tumorigenic. When cells cultured *in vitro* form colonies in soft agar suspension they will usually give tumours in syngeneic hosts. This test, therefore, can serve as a valuable assay system for detecting such tumorigenic cells in tissue culture.

It is interesting that P cultures contained a higher percentage of transformed cells than N cultures according to the assay results (Table I). This indicates that treatment of rat embryo cells with 25 $\mu$g/ml NMU tends to inhibit spontaneous transformation, perhaps by having a preferential killing effect on cells that would later have transformed spontaneously. However, tumours arising in rats from uncloned P and N cells do so with similar frequency after similar latent periods (Table II). We conclude that the agar assay is useful in qualitative studies of transformation. Further studies will determine whether it is equally useful in quantitative studies. It is also interesting that the cells should have spontaneously transformed so readily with bovine foetal serum (BFS) present in the medium since previous studies with C3H mouse embryo cells (Evans and Andresen, 1966; Sanford *et al.*, 1972) showed BFS to be an inhibitor of spontaneous transformation when compared with horse serum. However, the concentrations of BFS used were in excess of 5% (cf. 2%), and it is not known whether the factor present in BFS which inhibits transformation of mouse cells has any effect on rat cells; from our results it would appear not, certainly at a level in the medium of 2%.

Although skin tumours have been obtained by topical application of NMU (Graffi, Hoffmann and Schütt, 1967), attempts at inducing subcutaneous tumours from single injections of NMU have been unsuccessful (Kelly *et al.*, 1968). We are unable, therefore, to compare the subcutaneous fibrosarcomata induced by N cells with tumours induced *in vivo* by NMU. The frequency of spontaneous, subcutaneous fibrosarcomata in Wistar rats is very low (Crain, 1958; Ratcliffe, 1940) and since the morphology of such tumours in rats has not been illustrated, we are unable to say if the tumours induced subcutaneously by P cells are similar to those occurring spontaneously. The best comparisons are therefore drawn with subcutaneous mouse sarcomata induced by chemicals or transformed cells, or arising spontaneously.

With the exception of tumours arising from cloned N cells, the fibrosarcomata we observed for the most part resembled spontaneous subcutaneous sarcomata occurring in mice (Slye, Holmes and Wells, 1917; Dunn, Heston and Deringer, 1956), although peripheral acellular areas were not observed, presumably due to replacement by more rapidly growing cells, as Dunn *et al.* (1956) suggest, occurs when tumours are left *in situ* for any length of time after their first appearance.

Nettleship *et al.* (1943) injected C3H mice with syngeneic cells that had transformed spontaneously in tissue culture. The tumours that arose were morphologically similar to the fibrosarcomata described in the present paper. In more recent experiments, Franks, Chesterman and Rowlatt (1970) obtained similar mouse tumours by the inoculation of transformed syngeneic cell lines derived from a variety of cultured adult and embryonic organs. Of the 3 main categories of tumours they described, we were able to recognize 2 types (myxoid and leiomyomatous) but were unable to locate epithelioid areas in any of the sections examined.

The presence of giant cells in most of our rat tumours is not unusual since they were noted by Dunn *et al.* (1956) as occurring in many spontaneous mouse fibrosarcomata. Moreover, the predominance of uninucleate types in myxoid areas and multinucleate types in leiomyomatous areas compares with the findings of Franks *et al.* (1970) in their mouse tumours. Although we have observed muscle giant cells in rapidly growing transplanted tumours, most of our giant cells bear more resemblance to those occurring in chemically-induced tumours (Bonser and Orr, 1939).

After a long latent period, cloned N cells gave rise to tumours with a high proportion of giant cells compared with all other tumours in the series. Earle *et al.* (1943) found that cells treated *in vitro* with methylcholanthrene (MC) for long periods (184 and 406 days) gave rise to tumours with higher proportions of giant cells and longer latent periods than tumours derived from cells treated for shorter periods. A similar result has been achieved in our experiments by the cloning of cells treated for a short period with a carcinogen.

Although Franks *et al.* (1970) discussed the possibility that some of their mouse tumours resembled certain types of haemangiopericytoma, no examples were illustrated. One tumour (Fig. 4) shows features described by Stout (1949) as typical of human haemangiopericytoma, and is similar in morphology to 2 examples he illustrated. The tumour illustrated in Fig. 6, showing pronounced perivascular whorling, could also be described as a haemangiopericytoma since Stout (1949) suggests the possibility that pericytes, being of mesenchymal origin, may change character and become fibroblastic. It is clearly difficult to distinguish between a well vascularized fibrosarcoma and a pericytoma with fibroblastic pericytes, and we heed the advice of Willis (1967*a*) in taking the utmost caution in diagnosing the latter, especially since the vascular features were not retained on transplantation and could, therefore, have been of host origin.

Spontaneous subcutaneous vascular tumours have been observed in Wistar rats by Crain (1958) and in mice by Slye *et al.* (1917) and Dunn *et al.* (1956), who recorded low incidences of angiosarcoma and haemangioendothelioma respectively. Nettleship *et al.* (1943) found one example of an angiomatous tumour in those arising from MC-transformed mouse cells, but Bonser and Orr (1939) and Lewis (1939) found no such tumours in mice after subcutaneous injections of carcinogenic polycyclic hydrocarbons. One tumour arising from uncloned N cells (Fig. 7) exhibited a wave-like structure similar to 2 human neurofibromatous lesions described by Stewart and Copeland (1931), and was also similar in morphology to a polyoma virus-induced tumour of the hamster reported by Berman (1967). Willis (1967*b*) states that neurofibromata tend to lay down a lot of collagen and this rat tumour was no exception in this respect; however, in the absence of any obvious neurogenic origin, the diagnosis of neurofibroma cannot be substantiated. Nettleship *et al.* (1943) produced a tumour showing neurogenic pattern from MC-transformed mouse cells, but whether or not this was associated with nervous tissue is not recorded.

The atypical round cells (Fig. 8) seen in one tumour that changed in morphology on transplantation, closely resembled those described as " macrophage-type " by Ahlström and Jonsson (1962), in a virus-induced rat Rous sarcoma. In 1941, Doljanski and Tenenbaum reported round cells in Rous sarcoma cultures *in vitro* and observed reversible change to spindle type. The round cells shown in Fig. 8 may result from a similar change *in vivo*. In an attempt to test the possibility of this tumour being a virogenic rat Rous sarcoma, a mince of the primary tumour (which gave rise to the round cell tumour on transplantation) was inoculated into the wing web of 7-day old White Leghorn chicks (Ahlström and Jonsson, 1962). No tumours arose in 6 weeks.

Franks *et al.* (1970) referred to the histological similarity between some of their tumours derived from spontaneously transformed cells and virus-induced tumours. C-type particles have been found in spontaneously transforming mouse and rat cells (Franks and Wilson, 1970; Gazzolo *et al.*, 1971; Bergs *et al.*, 1972). It has been suggested that transformation of cells after chemical treatment occurs only if a C-type virus particle is present in the culture either by infection (Freeman *et al.*, 1970, 1971*b*; Price *et al.*, 1971; Rhim, Creasy and Huebner, 1971*a*; Rhim *et al.*, 1971*b*), or as a result of activation by the chemical (Freeman *et al.*, 1971*a*), in which case the virus can be detected in the tumour and in tumour-derived cell lines. Using our standardized search method for viruses with the electron microscope, we are satisfied that none of the transformed cells, tumour tissue or tumour-derived cell lines examined carried visible virus particles.

We would like to thank Professor R. J. C. Harris and the late Dr F. C. Chesterman for helpful discussion and criticism, and Dr D. Šimkovič for providing the WERC cells. We are also grateful for the excellent technical assistance of Mr G. B. Carter, Mrs D. Batter-Hatton and Mrs M. O. Phillips in the preparation of the histological sections, Mr A. B. Dowsett and Mrs R. Tilly for the electron microscopic investigation and Mr G. Leach for the photographs.

This work was carried out jointly at the Mill Hill laboratories of the Imperial Cancer Research Fund and the Microbiological Research Establishment, Porton, Nr Salisbury, Wilts. whilst the authors were holding ICRF bursaries for training in research.

### REFERENCES

Ahlström, C. G. & Jonsson, N. (1962) Induction of Sarcoma in Rats by a Variant of Rous Virus. *Acta path. microbiol. scand.*, **54**, 145.

Bergs, V. V., Pearson, G., Chopra, H. C. & Turner, W. (1972) Spontaneous Appearance of Cytopathology and Rat C-type Virus (WF–1) in a Rat Embryo Cell Line. *Int. J. Cancer*, **10**, 165.

Berman, L. D. (1967) Comparative Morphologic Study of the Virus-induced Solid Tumors of Syrian Hamsters. *J. natn. Cancer Inst.*, **39**, 847.

Bonser, G. M. & Orr, J. W. (1939) The Morphology of 160 Tumours Induced by Carcinogenic Hydrocarbons in the Subcutaneous Tissues of Mice. *J. Path. Bact.*, **49**, 171.

Crain, R. C. (1958) Spontaneous Tumors in the Rochester Strain of the Wistar Rat. *Am. J. Path.*, **34**, 311.

DiPaolo, J. A., Donovan, P. & Nelson, R. (1969*a*) Quantitative Studies of *in vitro* Transformation by Chemical Carcinogens. *J. natn. Cancer Inst.*, **42**, 867.

DiPaolo, J. A., Donovan, P. J. & Nelson, R. L. (1971*a*) X-irradiation Enhancement of Transformation by Benzo(a)pyrene in Hamster Embryo Cells. *Proc. natn. Acad. Sci. U.S.A.*, **68**, 1734.

DiPaolo, J. A., Nelson, R. L. & Donovan, P. J. (1969*b*) Sarcoma-producing Cell Lines Derived from Clones Transformed *in vitro* by Benzo(a)-pyrene. *Science, N.Y.*, **165**, 917.

DiPaolo, J. A., Nelson, R. L. & Donovan, P. J. (1971*b*) Morphological, Oncogenic, and Karyological Characteristics of Syrian Hamster Embryo Cells Transformed *in vitro* by Carcinogenic Polycyclic Hydrocarbons. *Cancer Res.*, **31**, 1118.

Doljanski, L. & Tenenbaum, E. (1941) Cellular Composition of Pure Rous Sarcoma Cultures *in vitro*. *Proc. Soc. exp. Biol. Med.*, **47**, 239.

Dougherty, R. M. (1962) Use of Dimethylsulphoxide for Preservation of Tissue Culture Cells by Freezing. *Nature, Lond.*, **193**, 550.

Dunn, T. B., Heston, W. E. & Deringer, M. K. (1956) Subcutaneous Fibrosarcomas in Strains C3H and C57BL Female Mice, and $F_1$ and Backcross Hybrids of these Strains. *J. natn. Cancer Inst.*, **17**, 639.

Earle, W. R., Schilling, E. L., Stark, T. H., Straus, N. P., Brown, M. F. & Shelton, E. (1943) Production of Malignancy *in vitro*. IV. The Mouse Fibroblast Cultures and Changes Seen in the Living Cells. *J. natn. Cancer Inst.*, **4**, 165.

Evans, V. J. & Andresen, W. F. (1966) Effect of Serum on Spontaneous Neoplastic Transformation *in vitro*. *J. natn. Cancer Inst.*, **37**, 247.

Franks, L. M., Chesterman, F. C. & Rowlatt, C. (1970) The Structure of Tumours Derived from Mouse Cells after " Spontaneous " Transformations *in vitro*. *Br. J. Cancer*, **24**, 843.

Franks, L. M. & Wilson, P. D. (1970) " Spontaneous " Neoplastic Transformation *in vitro*; The Ultrastructure of the Tissue Culture Cell. *Eur. J. Cancer*, **6**, 517.

Freeman, A. E., Kelloff, G. J., Gilden, R. V., Lane, W. T., Swain, A. P. & Huebner, R. J. (1971*a*). Activation and Isolation of Hamster-specific C-type RNA Viruses from Tumors Induced by Cell Cultures Transformed by Chemical Carcinogens. *Proc. natn. Acad. Sci. U.S.A.*, **68**, 2386.

Freeman, A. E., Price, P. J., Bryan, R. J., Gordon, R. J., Gilden, R. V., Kelloff, G. J. & Huebner, R. J. (1971*b*) Transformation of Rat and Hamster Embryo Cells by Extracts of

City Smog. *Proc. natn. Acad. Sci. U.S.A.*, **68**, 445.
FREEMAN, A. E., PRICE, P. J., IGEL, H. J., YOUNG, J. C., MARYAK, J. M. & HUEBNER, R. J. (1970) Morphological Transformation of Rat Embryo Cells Induced by Diethylnitrosamine and Murine Leukemia Viruses. *J. natn. Cancer Inst.*, **44**, 65.
GAZZOLO, L., ŠIMKOVIČ, D. & MARTIN-BERTHELON, M. C. (1971) The presence of C-type RNA Virus Particles in a Rat Embryo Cell Line Spontaneously Transformed in Tissue Culture. *J. gen. Virol.*, **12**, 303.
GORDON, H. & SWEETS, H. H. JR. (1936) A Simple Method for the Silver Impregnation of Reticulum. *Am. J. Path.*, **12**, 545.
GRAFFI, A., HOFFMANN, F. & SCHÜTT, M. (1967) N-methyl-n-nitrosourea as a Strong Topical Carcinogen when Painted on Skin of Rodents. *Nature, Lond.*, **214**, 611.
KELLY, M. G., O'GARA, R. W., YANCEY, S. T. & BOTKIN, C. (1968) Carcinogenicity of l-methyl-l-nitrosourea in Newborn Mice and Rats. *J. natn. Cancer Inst.*, **41**, 619.
LEWIS, W. H. (1939) Dibenzanthracene Mouse Sarcomas. Histology. *Am. J. Cancer*, **37**, 521.
MACPHERSON, I. & MONTAGNIER, L. (1964) Agar Suspension Culture for the Selective Assay of Cells Transformed by Polyoma Virus. *Virology*, **23**, 291.
NAMBA, M. & SATO, J. (1971) Carcinogenesis in Tissue Culture. XV. Aggregate-forming Capacity of Rat Liver Cells. Comparison of Untreated Controls, Cells Transformed in Culture, and Tumor Produced by Backtransplantation. *Jap. J. exp. Med.*, **41**, 233.
NETTLESHIP, A., EARLE, W. R., CLAPP, M. P. & SHELTON, E. (1943) Production of Malignancy *in vitro*. VI. Pathology of Tumors Produced. *J. natn. Cancer Inst.*, **4**, 229.
OSHIRO, Y., GERSCHENSON, L. E. & DIPAOLO, J. A. (1972) Carcinomas from Rat Liver Cells Transformed Spontaneously in Culture. *Cancer Res.*, **32**, 877.
OTSUKA, H. (1972) An Inhibitor Present in Calf Serum which Prevents Growth of BHK 21 Cells in Suspension Culture. *J. cell. Sci.*, **10**, 137.
PRICE, P. J., FREEMAN, A. E., LANE, W. T. & HUEBNER, R. J. (1971) Morphological Transformation of Rat Embryo Cells by the Combined Action of 3-methylcholanthrene and Rauscher Leukaemia Virus. *Nature, New Biol.*, **230**, 144.
RATCLIFFE, H. L. (1940) Spontaneous Tumors in Two Colonies of Rats at the Wistar Institute of Anatomy and Biology. *Am. J. Path.*, **16**, 237.
RHIM, J. S., CREASY, B. & HUEBNER, R. J. (1971*a*) Production of Altered Cell Foci by 3-methylcholanthrene in Mouse Cells Infected with AKR Leukemia Virus. *Proc. natn. Acad. Sci. U.S.A.*, **68**, 2212.
RHIM, J. S., VASS, W., CHO, H. Y. & HUEBNER, R. J. (1971*b*) Malignant Transformation Induced by 7,12-dimethylbenz(a)anthracene in Rat Embryo Cells Infected with Rauscher Leukemia Virus. *Int. J. Cancer*, **7**, 65.
SANDERS, F. K. & BURFORD, B. O. (1967) Morphological Conversion of Cells *in vitro* by n-nitrosomethylurea. *Nature, Lond.*, **231**, 1171.
SANFORD, K. K., JACKSON, J. L., PARSHAD, R. & GANTT, R. R. (1972) Evidence for an Inhibiting Influence of Bovine Fetal Serum on "Spontaneous" Neoplastic Transformation *in vitro*. *J. natn. Cancer Inst.*, **49**, 513.
SATO, J., NAMBA, M., USUI, K. & NAGANO, D. (1968) Carcinogenesis in Tissue Culture. VIII. Spontaneous Malignant Transformation of Rat Liver Cells in Long-term Culture. *Jap. J. exp. Med.*, **38**, 105.
SHARON, N. & POLLARD, M. (1969) Spontaneous Neoplastic Transformation of Germ-free Rat Embryo Cell Culture. *Cancer Res.*, **29**, 1523.
SLYE, M., HOLMES, H. F. & WELLS, H. G. (1917) Primary Spontaneous Sarcoma in Mice. Eighth Communication. *J. Cancer Res.*, **2**, 1.
STEWART, F. W. & COPELAND, M. M. (1931) Neurogenic Sarcoma. *Am. J. Cancer*, **15**, 1235.
STOUT, A. P. (1949) Hemangiopericytoma. A Study of Twenty-five New Cases. *Cancer, N.Y.*, **2**, 1027.
VESELÝ, P., DONNER, L. & KUČEROVÁ, M. (1968) Spontaneous Malignant Transformation of Embryonic Rat Fibroblasts from an Inbred Lewis Strain *in vitro*. *Folia biol., Praha*, **14**, 409.
WILLIS, R. A. (1967*a*) *Pathology of Tumours*. London: Butterworths. p. 733.
WILLIS, R. A. (1967*b*) *Pathology of Tumours*. London: Butterworths. p. 664.